# The emerging role of *Toxoplasma gondii* in periodontal diseases and underlying mechanisms

**DOI:** 10.3389/fimmu.2024.1464108

**Published:** 2024-10-03

**Authors:** Henglong Cao, Jianfeng Lin, Hao Yuan, Zipeng Yang, Min Nie, Janak L. Pathak, Zi-Guo Yuan, Miao Yu

**Affiliations:** ^1^ Department of Periodontics, School and Hospital of Stomatology, Guangdong Engineering Research Center of Oral Restoration and Reconstruction, Guangzhou Key Laboratory of Basic and Applied Research of Oral Regenerative Medicine, Guangzhou Medical University, Guangzhou, China; ^2^ Guangdong Provincial Key Laboratory of Zoonosis Prevention and Control, College of Veterinary Medicine, South China Agricultural University, Guangzhou, China; ^3^ Department of Oral Health Sciences-BIOMAT, KU Leuven and Dentistry, University Hospitals Leuven, Leuven, Belgium

**Keywords:** *T. gondii*, periodontitis, pathogenic mechanisms, immune response, inflammation, cytokines

## Abstract

*Toxoplasma gondii* (*T. gondii*), an obligate intracellular protozoan parasite, is increasingly recognized for its role in various human diseases, including periodontal diseases. Periodontal diseases comprise a wide range of inflammatory conditions that not only affect the supporting structures of the teeth and oral health but also contribute to systemic diseases. The parasite’s ability to modulate the host’s immune response and induce chronic inflammation within the periodontium is a key factor in periodontal tissue damage. Through its virulence factors, *T. gondii* disrupts the balance of inflammatory cytokines, leading to dysregulated immune responses, and exacerbates oxidative stress in periodontal tissues. And *T. gondii* invasion could affect specific proteins in host cells including HSP70, BAGs, MICs, ROPs, SAGs, and GRAs leading to periodontal tissue damage. The indirect role of the host immune response to *T. gondii* via natural killer cells, monocytes, macrophages, neutrophils, dendritic cells, T cells, and B cells also contributes to periodontal diseases. Understanding these complex interactions of *T. gondii* with host cells could unravel disease mechanisms and therapeutic targets for periodontal diseases. This review delves into the pathogenic mechanisms of *T. gondii* in periodontal diseases, offering a detailed exploration of both direct and indirect pathways of its impact on periodontal health.

## 1 Introduction

Periodontitis is a chronic infectious disease that affects the periodontal support tissues including the gingiva, bone, and periodontal ligament, and could lead to tooth loss. The prevalence of periodontitis is high, with reported rates ranging from 17.60% to 90.00% in different regions around the world (1, 2). Periodontitis is a multifactorial disease, and the exact cause is not yet clear. Plaque biofilm serves as the initiating factor, and the unique periodontal microenvironment is formed through the interaction among microorganisms, hosts, and the environment in the periodontal area. The imbalance of these interactions destroys periodontal tissue (3–5). The classic theory of periodontitis etiology suggests that periodontitis is caused by an increase in non-specific oral microbiota (6). Changes in the local microenvironment can result in a decrease in beneficial bacteria and an increase in pathogenic bacteria, potentially leading to chronic diseases like periodontitis. Based on the theory above, the traditional treatment method for periodontal diseases is still mechanical plaque removal (7). However, the current theory of plaque pathogenesis is unable to fully explain all the clinical features of periodontal diseases. Treatment approaches rooted in this theory have minimal impact on advanced periodontal diseases. The role of this treatment method in preventing chronic periodontal diseases is also very limited (8, 9).

With the progress of research methodologies and technological advancements, the investigation into the etiology of periodontitis has progressively gained more depth. In recent years, scholars have proposed new models and concepts to supplement the traditional etiology theory to enhance the understanding of the pathogenesis of periodontitis. The changes in the host’s health status are attributed to the interactions among these microorganisms, as well as their interactions with the host, which are inherently influenced by the host’s internal and external environment. All living microorganisms possess intricate communities comprising viruses, bacteria, and other microorganisms, including parasites, which have the potential to interact and influence their health and disease status at any given moment. Inspired by this, parasites have garnered increasing attention as a potential pathogenic factor for periodontal diseases, in addition to bacteria and viruses. *T. gondii* is an opportunistic pathogenic parasite and can cause zoonotic toxoplasmosis (10, 11). A survey has found that one-third of the world’s population is infected with *T. gondii* (12, 13). The infection rate of *T. gondii* in animals varies greatly i.e., 10.45% to 66.47% in pigs, 2.50% to 60.00% in cats, and 0.56% to 27.65% in dogs (14, 15). The primary routes of human infection are through consuming raw poultry or water that contains *T. gondii* oocysts. After *T. gondii* invades the human body, it mainly causes a latent infection, primarily distributed in the brain and muscle tissue (16). However, for individuals with immune deficiency, severe symptoms can occur, such as encephalitis and even death (11). After infection with *T. gondii*, it can spread through the bloodstream and reach different organs, causing damage to the brain, heart, and other parts of the body (3). So far, there is no effective vaccine or drug available to prevent toxoplasmosis. *T. gondii* can cause latent infections, activate certain conditions, and subsequently alter the inflammatory response of cells. This pathogenic pattern is very similar to the characteristic of paroxysmal accelerated destruction seen in certain specific periodontal diseases.

In recent years, there has been growing attention to the relationship between *T. gondii* infection and oral diseases. Currently, studies have shown that the primary parasites linked to periodontal diseases are *Entamoeba gingivalis*, *Trichomonas tenax*, and *T. gondii* (15, 16). The interaction between the oral microbiome *and T. gondii* could contribute to the development and progression of periodontal diseases including periodontitis. This review aimed to summarize the possible virulence of *T. gondii* in periodontal tissues, and its role in the development and progression of periodontal diseases. We also discussed the *T. gondii*-related additional perspective in the etiology of periodontal diseases that could provide novel strategies for prevention and treatment.

## 2 *T. gondii* and human diseases

Foodborne diseases are a significant public health concern, affecting millions of people worldwide (2). Among these, parasitic infections transmitted through contaminated food or water are particularly concerning. Parasites can cause a range of diseases, including those that affect the gastrointestinal tract, as well as systemic infections that can lead to serious complications (10). One such parasitic infection that can be acquired through the oral route is toxoplasmosis, caused by the ubiquitous opportunistic pathogen *T. gondii*.

**Figure 1:**
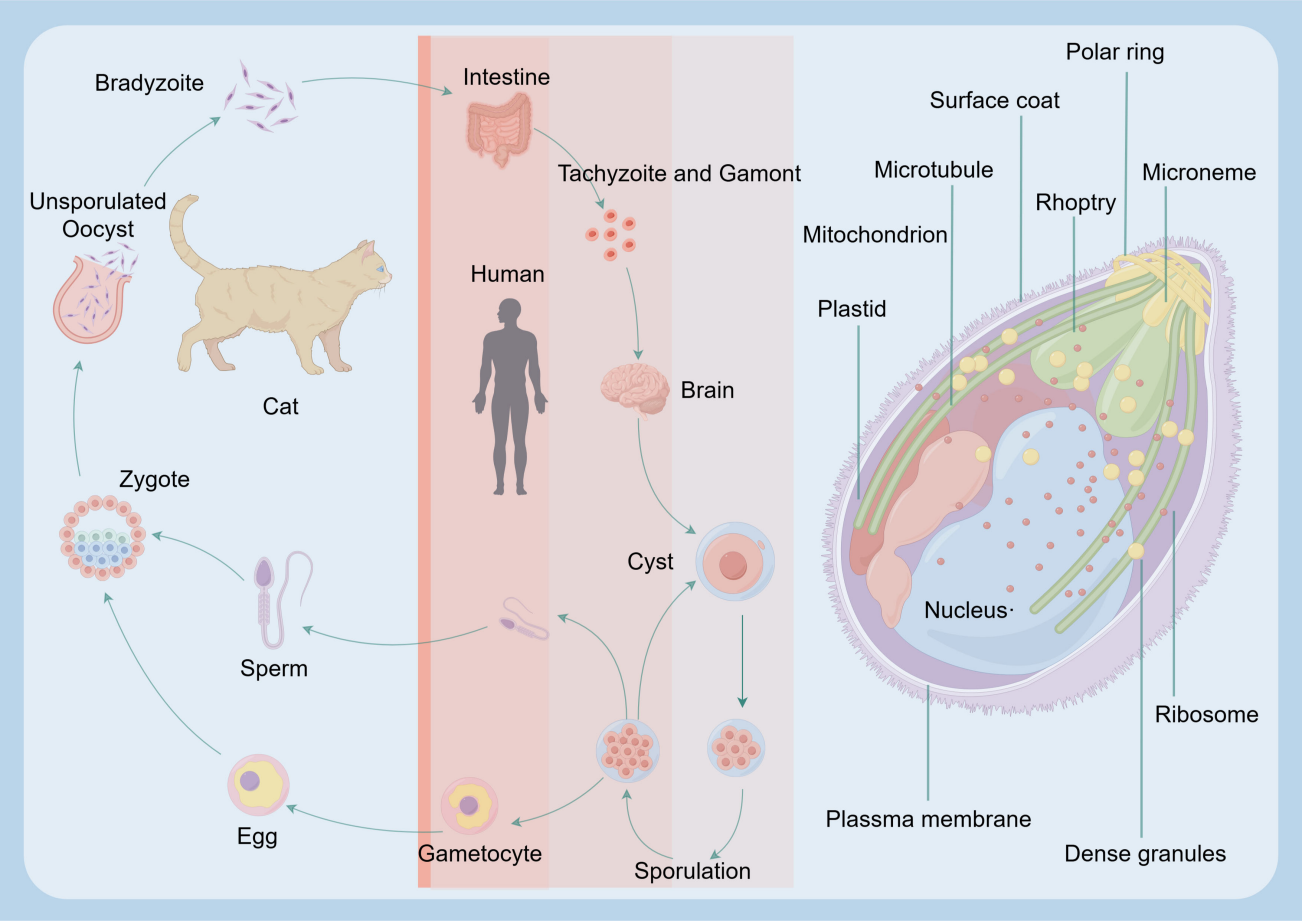
Major routes *T. gondii* infection in humans. The reproductive cycle of *T. gondii* begins when a member of the feline family ingests either oocysts or tissues infected with bradyzoite cysts. This cycle occurs within the feline intestine and results in the excretion of oocysts in the cat’s feces. Following oocyst maturation, the oocysts become highly infectious. Any warm-blooded animal that ingests these infectious oocysts becomes a host for the asexual cycle. Upon release from the oocyst, the sporozoites will infect the intestinal epithelium and differentiate into the tachyzoite stage. After an acute infection, characterized by the spread of tachyzoites throughout the body, tissue cysts develop as a result of differentiation into the bradyzoite stage. Upon ingesting these tissue cysts in raw or undercooked meat from a chronically infected host, the bradyzoites will infect the intestinal epithelium of the next susceptible host and differentiate into the tachyzoite stage to complete the asexual cycle. If the ingesting animal is a cat, the bradyzoites can differentiate into the sexual stages, completing the entire life cycle.

**Figure 2:**
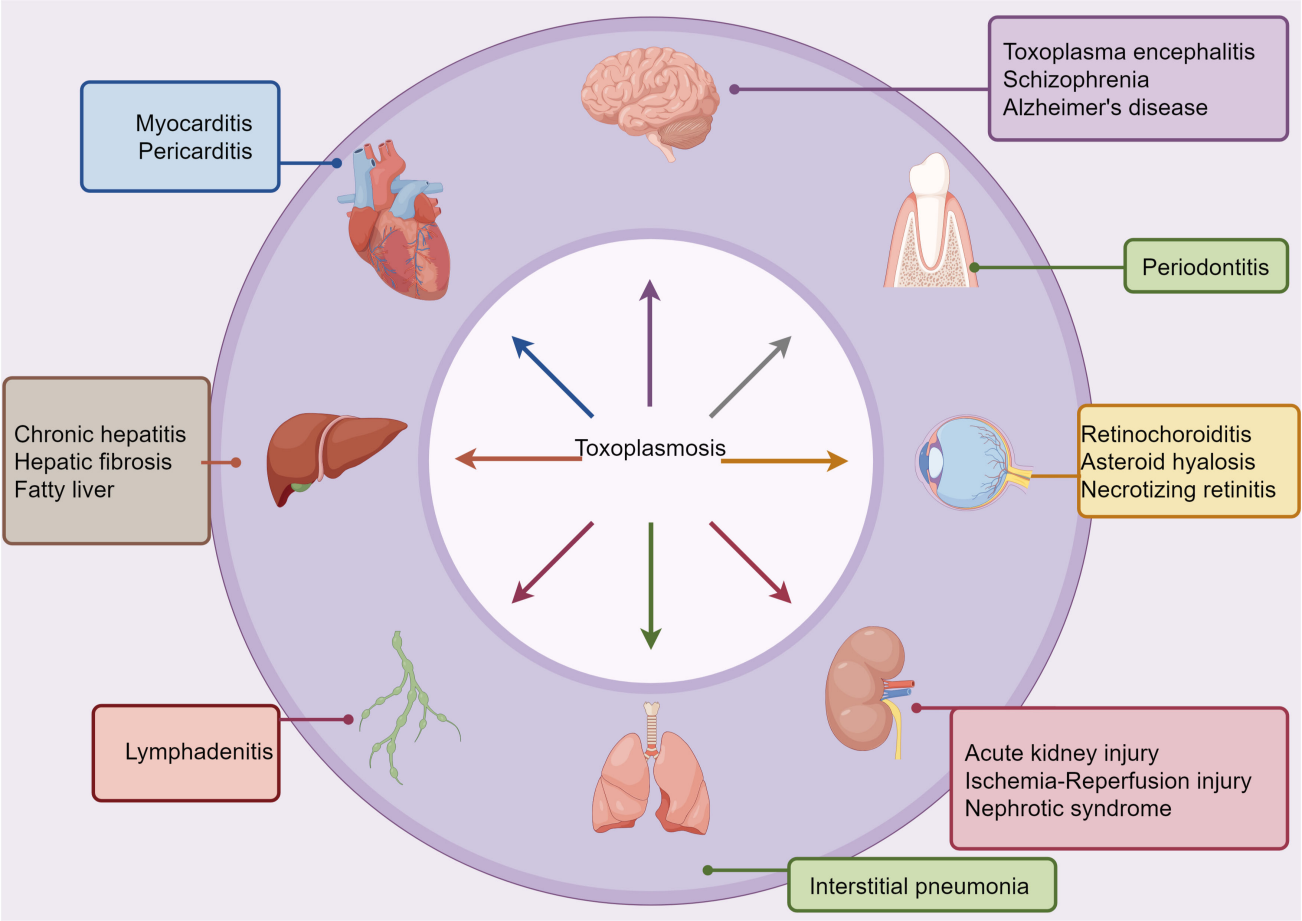
Links between *T. gondii* infection and systemic diseases. Based on epidemiological, clinical intervention, and animal model-based studies, *T. gondii* infection has been associated with several comorbid conditions, as indicated. *T. gondii* can spread through various routes, including hematogenous, oropharyngeal, and digestive pathways, to reach extraoral sites where they can cause or worsen inflammatory pathologies.

*T. gondii* is an opportunistic pathogen that is widely distributed worldwide. It needs animals or humans to complete the life cycle (**Figure 1**). It can cause toxoplasmosis, posing a severe threat to human health (**Figure 2**) (15). The infection of *T. gondii* in individuals with normal immune function often results in latent *T. gondii* infection without obvious clinical symptoms. However, in individuals with immune deficiency, such as those with malignant tumors and AIDS, the infection can lead to severe consequences, including encephalitis, ophthalmic complications, and damage to other organs (17). The pathogenicity of *T. gondii* is primarily determined by its virulence, with the host’s immune status also playing a significant role. Therefore, the interaction between *T. gondii* and its host determines the severity of toxoplasmosis. The main stage of *T. gondii* pathogenesis is the release of tachyzoites and free trophozoites from pseudocysts (18). As the number of bradyzoites increases during the proliferation process, they can compress the organs and lead to functional disorders. When the proliferation of cysts reaches a certain level, several factors can cause the cysts to rupture. After the rupture, the released worms can stimulate the body and cause delayed allergic reactions, while forming granulomatous lesions, which are often found in the eyes, brain, and other areas. Under normal circumstances, when the host is infected with *T. gondii*, the body can produce effective immune protection, and usually, there are no obvious symptoms. Toxoplasmosis can only occur when the host has immune deficiency or depression (10, 18). AIDS patients may suffer from *Toxoplasma* encephalitis and may periodically experience associated diseases (19).

## 3 *T. gondii* and periodontal diseases

In recent years, there has been growing attention to the relationship between *T. gondii* infection and oral diseases. Gingival bleeding is a common symptom of periodontitis, creating favorable conditions for oral infection by *T. gondii*. With the advancement of molecular biology, more studies have revealed a strong correlation between parasites and oral diseases, as well as systemic diseases (12–14). Currently, studies have shown that the primary parasites linked to periodontal diseases are *Entamoeba gingivalis*, *Trichomonas tenax*, and *T. gondii* (15, 16). We speculate that there is an interaction between *T. gondii* and periodontal pathogens in the development of periodontitis. The mechanisms include the attachment and colonization of periodontal pathogens, the activation of latent *T. gondii*, and the suppression of the host immune response against periodontal pathogens. On one hand, parasitic infection reduces the host’s resistance, leading to abnormalities in local immune function. This increases the risk of bacterial infection and facilitates the colonization of periodontal pathogens (17). On the other hand, periodontal inflammation caused by bacteria benefits parasites by enabling them to infect cells and enter gingival tissue or the bloodstream. Further research is needed to elucidate the role of pathogen interactions and the associated host reactions with *T. gondii* during the development and progression of periodontitis.

## 4 The invasion, migration, and persistent infection process of *T. gondii* in host tissues

**Figure 3:**
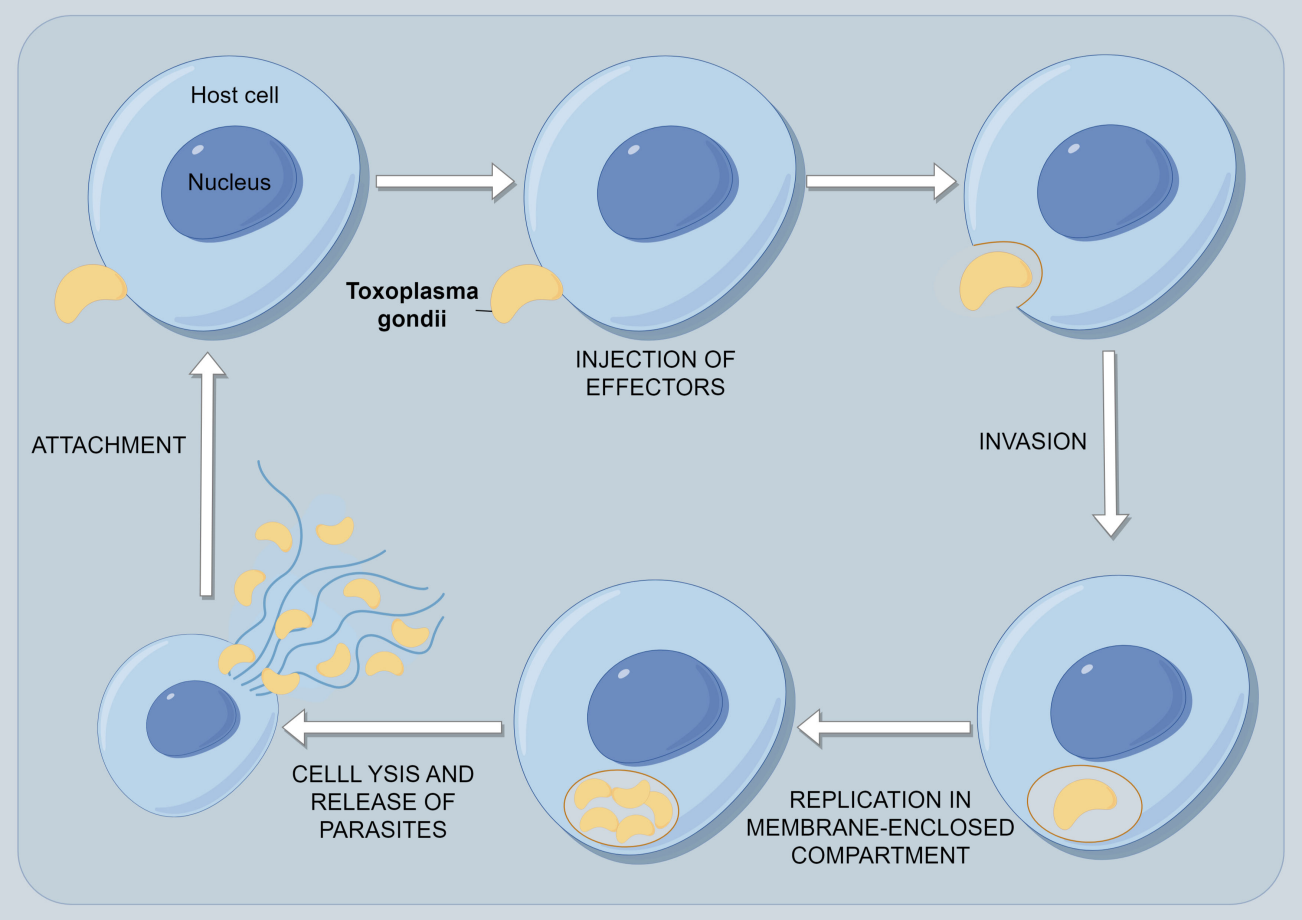
The process of persistent infection of *T. gondii* within host cells. The first event is the attachment of *T. gondii* to its host cell. As the parasite glides across the surface, it reorients itself to make contact with the host surface at its apical end and initiate invasion. During the invasion event, the PV is formed and modified by secretions from both the rhoptries and, later, the dense granules. After the parasite enters its host, it ceases movement, and the vacuole closes at its posterior end by pinching off through a fission pore. The newly formed PV immediately recruits host mitochondria and portions of the host endoplasmic reticulum. The parasites undergo multiple rounds of replication until they receive a signal from either the parasite itself or the host cell, leading to egress. Host cell egress is usually a destructive process that causes the host cell to lyse and release motile parasites. These parasites quickly invade neighboring cells to complete their life cycle.

*T. gondii* can invade and replicate intracellularly in virtually all nucleated cell types of the host body. The invasion of host cells by *T. gondii* is an active process, including attachment and invasion to host cells, formation of parasitophorous vacuoles (PV) within host cells, and modification of PV (**Figure 3**) (3, 18, 19). The subcellular organelles at the top of the *T. gondii* body have a secretory function, and the microtome can secrete a large number of microtome proteins (20). These proteins bind to the host cell membrane and adhere to the host cell. The rhoptry can secrete rod-shaped neck proteins (RONs) and also adhere to the host cell membrane (20, 21). These two types of proteins form a junction region between the front end of the parasite and the host cell through specific reactions (22). The parasite relies on its unique actin-myosin motor structure under the protoplasmic membrane to quickly squeeze through the junction region and enter the host cell (23). The proteins secreted by the Rhoptry and dense granule antigens (GRAs) secreted by the dense granules jointly form a parasitophorous vacuole membrane (PVM) to envelop the body of *T. gondii*. At the same time, it secretes rhomboid proteases to cleave the junction region and invade the host cell successfully (24). Finally, it modifies the components of the PVM to prevent phagocytosis of the host lysosome. Some studies suggest that certain immunosuppressive substances produced by *T. gondii* after invading host cells can disrupt the host’s overall immune system, evade recognition by neutrophils and phagocytes, and thus evade the host’s immune response (25). At the same time, when the host immune system responds to the antigens of cells infected with *T. gondii* but is unable to clear its cells, continuous immune attacks on periodontal tissue may happen, ultimately leading to periodontal diseases.

**Figure 4:**
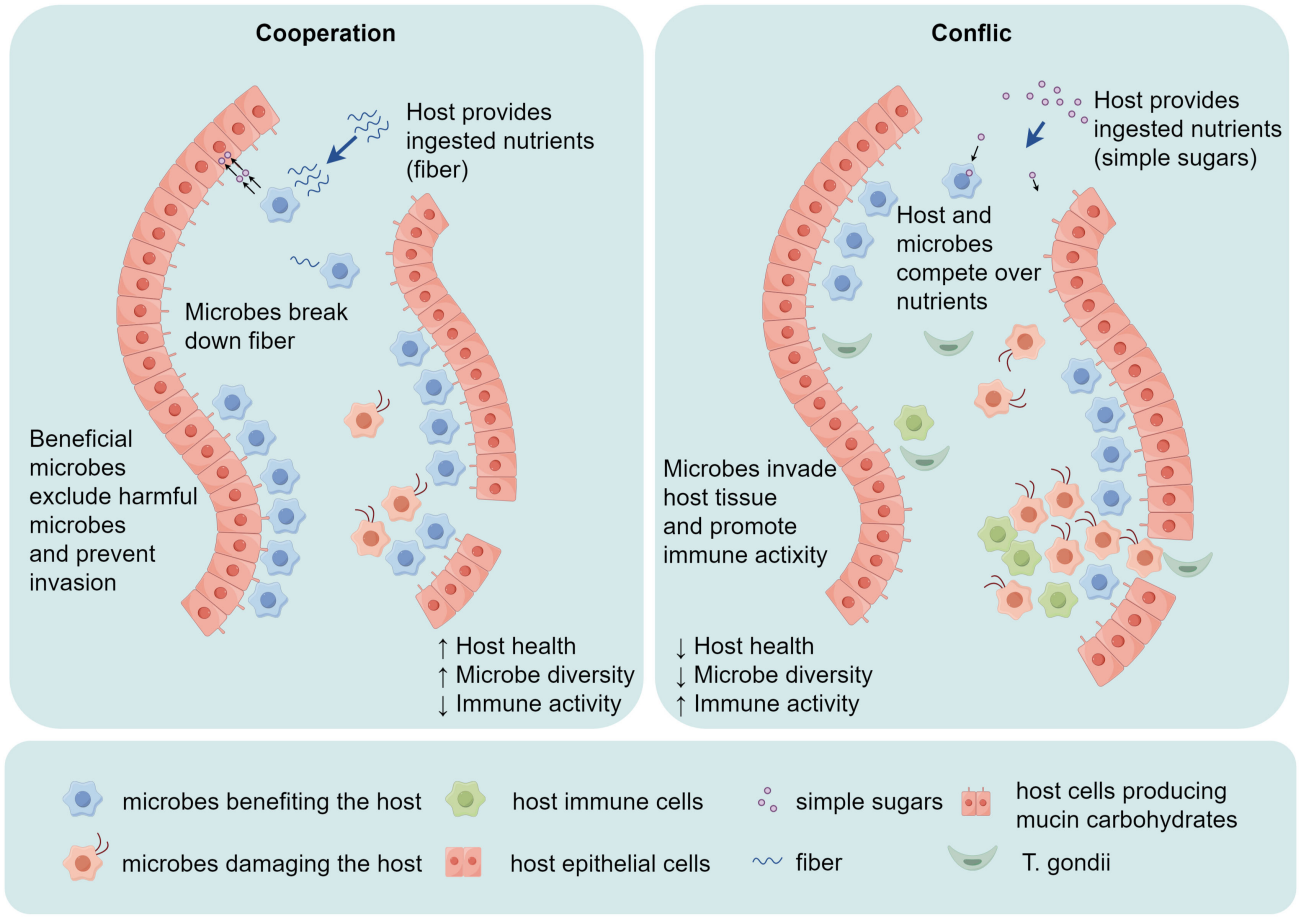
*T. gondii* invades the host’s intestinal mucosa, causing alterations in gut microbial diversity and the host’s immune activity. In a healthy state, there is a balance between local challenge and a mild host immune response. Both the commensal microbiota and host cells contribute to the development of local mucosal immunity. However, if the immune pathogenicity of the local microbiota is heightened by the invasion of *T. gondii*, which overactivates the host immune response, tissue destruction is initiated.

After invasion into the host, *T. gondii* tachyzoites will spread to different organs and pass through some physiological barriers, such as the gut, blood-brain barrier, and placenta. During the migration process, *T. gondii* tachyzoites first gather around cell junctions, possibly crossing physiological barriers through a paracellular pathway. Because during the migration process of *T. gondii*, the host cell membrane remains intact, and the secretion of intercellular adhesion molecule-1 (ICAM-1) on the host cell surface is upregulated. The expression of ICAM-1 is upregulated in human retinal pigment epithelial cells and retinal endothelial cells after *Toxoplasma gondii* infection. *In vitro* studies have shown that tachyzoite-infected dendritic cells (DCs) can cross the retinal endothelium with the assistance of ICAM-1, thus anti-ICAM-1 antibodies can prevent *T. gondii* migration. These results indicate that ICAM-1 plays a vital role in the migration process of *T. gondii* (26). After entering the host cells, *T. gondii* initially manipulates the host’s immune response by interfering with the transcription of nuclear factor kappa-B (NF-κB) and the activation of mitogen-activated protein kinase (MAPK). This interference temporarily suppresses the production of key pro-inflammatory cytokines, such as interleukin-12 (IL-12) and tumor necrosis factor-α (TNF-α). However, *T. gondii* also induces a strong type 1 response, characterized by high production of IL-12 and Interferon-γ (IFN-γ), which are crucial for controlling the parasite. Subsequently, the parasite employs evasion mechanisms to establish a persistent infection while the immune system remains effective in most hosts (27). As shown in **Figure 4**, *T. gondii* can disrupt the host’s immune response, cause intestinal dysbiosis, and greatly promote its migration. Therefore, we could speculate that *T. gondii* can either directly enter the bloodstream, causing lesions, or it may invade the host’s intestinal mucosa spread within the host’s body, and finally enter the host’s periodontal tissue, causing corresponding damage (28, 29).

To survive in the host for a long time and sustain the infection, *T. gondii* can alter the apoptosis process of its parasitic cells and nearby uninfected cells (30). After *T. gondii* infection, T cells and other white blood cells are induced to apoptosis. To prevent the process of apoptosis of infected host cells, *T. gondii* employs several mechanisms. The secretion of numerous parasite-effector proteins is a key process during infection. These effectors regulate host transcription, deregulating the host expression profile to facilitate host cell transformation or escape from the host immune system, allowing parasite persistence and survival (24). Induction of a host epigenetic enzyme, such as SMYD3, a methyltransferase that activates genes involved in host transformation through H3K4 trimethylation. Secretion of effector proteins, like TEEGR, that drive epigenetic repression of host genes through H3K27 trimethylation. Parasites target host chromatin regulatory complexes, where TgIST sequesters STAT1 and interacts with the NuRD complex, leading to a repressed chromatin state; regulating signaling by molecular mimicry, exemplified by TaPin1 degrading FBW7, stabilizing c-Jun and upregulating oncogenic genes. Parasite-secreted transcripts target host epigenetic enzymes, such as Cdg7 interacting with PRDM1/G9a in the host nucleus, leading to the repression of host immunity genes through H3K9 trimethylation. Moreover, the parasite induces host microRNAs, like miR-126, that target host regulatory pathways, releasing JNK1 and leading to phosphorylation of c-Jun and activation of genes involved in host transformation. Through the above-mentioned strategies, *T. gondii* suppresses the immune response just enough to avoid clearance while still permitting the host to maintain a certain level of immune surveillance, maintaining a delicate balance essential for the long-term survival of both the host and the parasite (31). Periodontitis is the result of a complex ecosystem composed of host immune, oral symbiotic microorganisms, pathogenic microorganisms, and environmental interactions (32, 33). Plaque biofilm plays an essential role in the occurrence of periodontitis, as it can damage the host’s immune system. Moreover, the interactions between various bacteria in the plaque biofilm also help it survive in the host and destroy periodontal tissue. Similarly, *T. gondii* can also suppress the host’s immune, thereby evading the host’s immune response. Therefore, in-depth research on the immune response of periodontal tissue and the invasion, migration, and persistent infection process of *T. gondii* can help deepen understanding of the pathogenesis of periodontal diseases and provide new ideas for its treatment and prevention.

## 5 *T. gondii*-related proteins could damage host periodontal tissue

*T. gondii* and its expressed proteins can directly damage periodontal tissue, or cause inflammatory reactions in periodontal tissue. The invasion of *T. gondii* is not achieved by a single protein or a single mechanism but by the cooperation of many proteins through multiple regulatory mechanisms. Among them, *T. gondii* heat shock protein 70 (Tg HSP70), bradyzoite antigens (BAGs), microneme proteins (MICs), rhoptry proteins (ROPs), surface antigens (SAGs), and GRAs all play essential roles in the active invasion of host cells by *T. gondii*, as shown in **Table 1** (24).

**Table 1:** *T. gondii*-related proteins-effect on host cells.

*T. gondii* related proteins	Study model	Immune regulation	Mechanisms	References
Tg HSP70	*In vivo*, gene-deficient mice	Anti-inflammatory	causes the host to produce Th2 cytokines such as IL-4 and IL-10	(35–37)
BAGs	*In vivo*, specific pathogen-free (SPF) mice	Immune activation	promote the differentiation of tachyzoites into bradyzoites, induce dendritic cells, and activate cellular immune responses	(18, 22, 38)
MICs	*In vitro*, macrophages	Immunomodulation	recognize and adhere to the host cell, and form a mobile junction structure	(20, 39)
ROPs	*In vitro*, macrophages	Anti-inflammatory	prevent the phagocytosis and clearance of the host body by macrophages	(21, 22, 41)
SAGs	*In vivo*, injection in mice	Immunomodulation	mediate the initial recognition and adhesion of the parasite to host cells	(38, 42, 43)
GRAs	*In vivo*, SPF mice	Immunomodulation	ensure the survival and replication of the parasite within the host cell by regulating the function of the host cell	(26)

The Tg HSP70 expressed in the tachyzoites causes the host to produce cytokines such as IL-4 and IL-10, which modulate the immune response (35, 36). These cytokines are known to shift the immune response away from a type 1 response, which is critical for controlling *T. gondii* infection, toward a type 2 response and immunoregulation, respectively. Czarnewski found that immunized mice with Tg HSP70 presented a significantly reduced number of cysts in the brain that was associated with increased iNOS^+^ cell numbers in the organ (37). BAG1 plays a vital role in the life cycle of *T. gondii*. The BAG1 gene can be used as a candidate antigen for DNA vaccines and induce protective immune responses. Recent research reported that DNA vaccine encoding BAG1 enhanced humoral and cellular immune responses effectively, and prolonged the survival time of infected mice (38). MICs play an essential role in the invasion of host cells. The apical secretory organelles of the *T. gondii* can secrete MICs, which recognize and adhere to the host cell, and form a moving junction structure (39). Rhoptry is also one of the three apical secretory organelles of *T. gondii*. ROPs are closely related to the virulence of the parasite. They participate in the invasion process and are involved in the PV. ROPs are mainly divided into two types, namely RONs and ROPs. The RONs are related to the formation of moving junctions, while the ROPs are involved in the formation and modification of PV (40). When *T. gondii* comes into contact with the host cell membrane, the rhoptry secretes a large number of RONs (such as RON2, RON4, RON5, and RON8), and the MICs form a tightly connected structure between the host cell membrane and the plasma membrane of *T. gondii*, known as moving junction (41). SAGs mainly mediate the initial recognition and adhesion of the parasite to host cells. In the discovered SAGs family, the primary proteins related to invasion and virulence are SAG1, SAG2, and SAG3 (42). Among them, SAG1 is the surface antigen with the highest expression level in *T. gondii* tachyzoites. In addition, SAG2 and SAG3 can assist SAG1 in rapidly invading the host cell. Previous studies have shown that these SAGs may be used as candidate antigens for the development of an anti-toxoplasmosis vaccine (38, 43). GRAs play an essential role in maintaining the structure of PVM. At the same time, GRAs also ensure the survival and replication of the parasite within the host cell by regulating the function of the host cell (26).

Recent studies have highlighted the role of *T. gondii* in activating the inflammasome and causing neuronal injury, which can indirectly contribute to the pathogenesis of periodontal diseases. *T. gondii* infection has been shown to activate the inflammasome, leading to the production of pro-inflammatory cytokines such as IL-18 and IL-1β (44). This activation is induced by specific *T. gondii* proteins, including profilin, which triggers the NLRP3 inflammasome pathway. The activation of the inflammasome leads to the production of IL-18, which is known to have a significant impact on the immune response. In a recent study by Andreou, *T. gondii* seropositivity was associated with elevated levels of IL-18 and neuron-specific enolase in both patients with severe mental illness and healthy controls (44). This finding suggests that *T. gondii* infection can cause ongoing inflammasome activation and neuronal injury, which may have broader implications for systemic inflammation and disease processes.

The inflammasome activation and neuronal injury caused by *T. gondii* infection can indirectly contribute to the pathogenesis of periodontal diseases. Chronic inflammation and dysregulated immune responses, which are hallmarks of periodontal diseases, are exacerbated by the release of pro-inflammatory cytokines such as IL-18 (32). This cytokine has been shown to play a crucial role in the development and progression of periodontal diseases by promoting the recruitment of inflammatory cells and increasing the production of matrix metalloproteinases (3), which are responsible for tissue destruction.

## 6 The possible role of host immune response induced by *T. gondii* in the pathogenesis of periodontal diseases

Periodontitis is a chronic inflammatory state that leads to the destruction of hard and soft tissues that support teeth (45). Its etiology involves various factors, such as periodontal pathogens, local promoting factors, genetic factors, and smoking habits. The development of periodontitis is not only caused by periodontal pathogens but also induced by the host’s immune inflammatory response (46). Therefore, dysregulation of signaling pathways is a potential mechanism for the onset of periodontitis (44). With in-depth research on the pathogenesis of periodontal diseases, we have realized that periodontal diseases are not only directly caused by the pathogen itself but indirectly caused by the host’s immune response to pathogens and their toxic products (32). Therefore, we speculate that periodontal diseases may be a local manifestation of a systemic immune response after host infection with parasites. The host’s immune response to parasites and their products, such as white blood cells, complement, antibodies, and cytokines can lead to periodontal tissue damage.

Inherent immune pattern recognition receptors associated with *T. gondii* play an essential role in periodontal diseases. Pattern recognition receptors have multiple family members, including the Toll-like receptor family (TLRs) (47). TLRs can perceive pathogen-associated molecular patterns (PAMPs) and activate host immune responses. So far, 10 TLRs (TLR1~TLR10) have been found in humans, and 12 TLRs in mice. Each TLR resides in a specific part of the cell and can perceive different PAMPs (48). In mice, TLR11 and TLR12 are the main effectors that can perceive *T. gondii*, expressed in the intracellular lysosomal membrane, and recognize *T. gondii* profilin proteins (49). In the TLR signaling pathway, myeloid differentiation primary response protein 88 (MyD88) is a receptor molecule for various TLRs and cytokines. After TLR11 activation, MyD88 is recruited to initiate downstream signaling and promote the secretion of IL-12 by dendritic cells to control the infection of *T. gondii* effectively (49). In the stage of chronic infection, the expression of TLR11 in astrocytes and microglia in brain tissue increases to facilitate the induction of immune response against *T. gondii* encephalitis (50). In human immune cells, TLR11 does function as a pseudogene, and TLR12 does not exist in the human genome (51). Qallaf found that assessments of salivary sTLR2 and sTLR4 together with the respective transcripts in the epithelial cells could provide clinically relevant markers of disease progression from gingivitis to periodontitis (52). Understanding how TLRs recognize PAMPs and trigger immune responses is crucial for understanding their role in *T. gondii*-associated periodontal diseases. For instance, TLRs recognize specific PAMPs, such as profilin via TLR11, which activates downstream signaling pathways leading to the production of proinflammatory cytokines and chemokines (49). This response helps to coordinate the innate and adaptive immune defenses against *T. gondii* and is critical for the control of the parasite and the prevention of periodontal tissue damage. Specifically, TLR11 recognition of *T. gondii* profilin leads to the activation of the MyD88-dependent pathway, which triggers the production of IL-12 by dendritic cells. This cytokine is essential for the differentiation of naive CD4^+^ T cells into IFN-γ-producing TH1 cells, which are critical for the control of *T. gondii* infection (49). Additionally, TLR2 and TLR4 recognize PAMPs derived from *T. gondii*, such as glycosylphosphatidylinositol-anchored proteins, contributing to the overall immune response against *T. gondii* (34). The recognition of these PAMPs by TLRs activates the NF-κB and MAPK signaling pathways, leading to the production of pro-inflammatory cytokines, including TNF-α and IL-6, which are important for the recruitment and activation of immune cells at the site of infection (48). Furthermore, TLR activation can also trigger the inflammasome pathway, leading to the maturation and secretion of IL-1β and IL-18, which are critical for the induction of TH1 cell responses and the production of IFN-γ (49). Together, these mechanisms highlight the multifaceted role of TLRs in recognizing *T. gondii* and coordinating the immune response to prevent periodontal tissue damage.

## 7 The role of immune cells and cytokines associated with periodontitis in the fight against *T. gondii*

**Figure 5:**
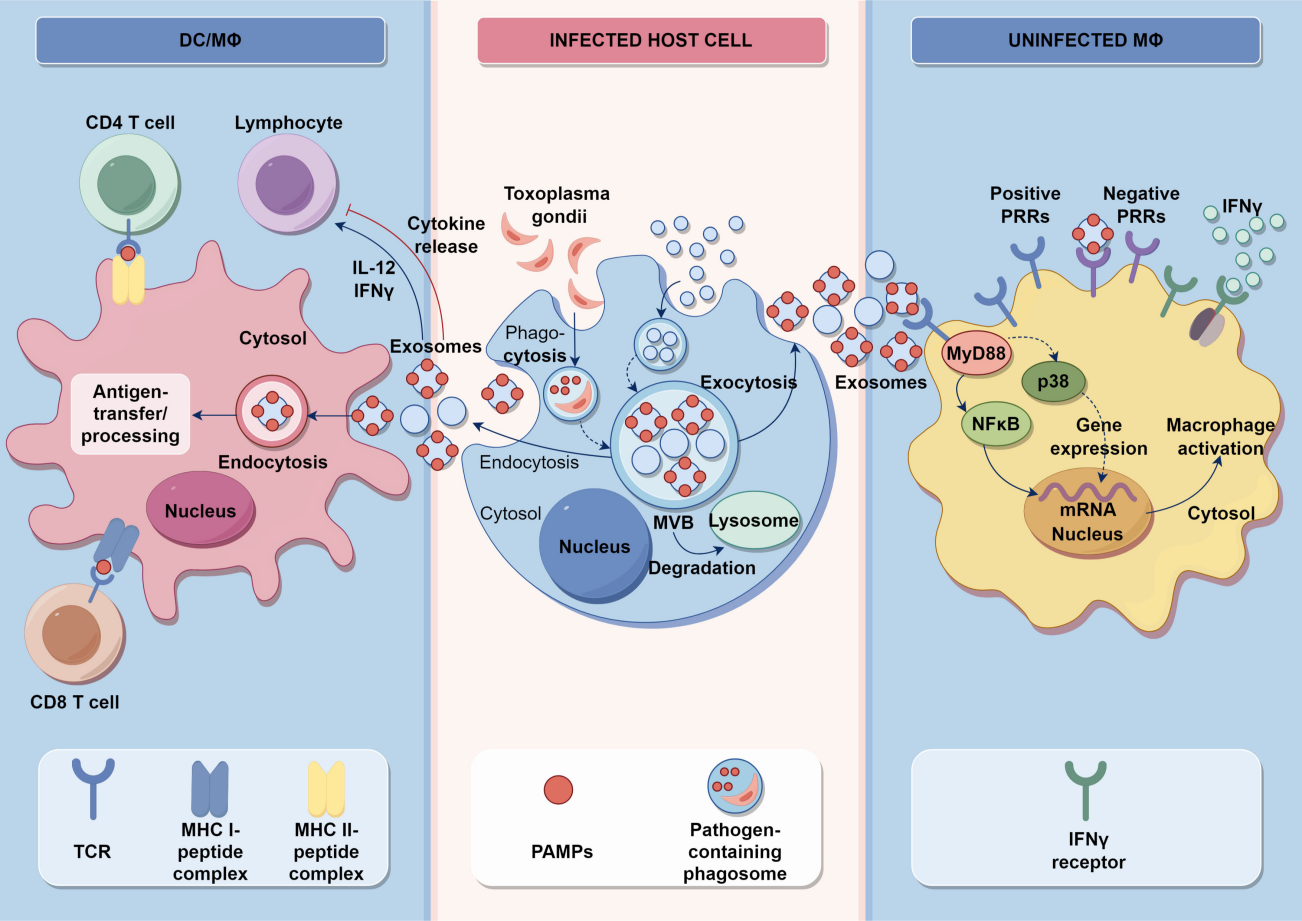
Innate and acquired immune signaling and the influence of *T. gondii* pathogen. Activation of intracellular TLRs leads to the production of IFNs and pro-inflammatory cytokines, creating an environment that is not conducive to pathogens. DCs and macrophages are stimulated by a variety of factors, including environmental stress or infection, to present antigens that lead to the differentiation of CD4^+^ T cells and CD8^+^ T cells. Additionally, they release proinflammatory cytokines such as TNF-α, IL-1β, and IL-6. These infected cells also produce exosomes that regulate the functionality of DCs and macrophages.

After infection with *T. gondii*, the immune status of the body significantly affects its outcome, primarily as cellular immunity is closely related to the prognosis of the disease (**Figure 5**). The immune cells associated with periodontitis mainly include natural killer (NK) cells, monocytes, macrophages, DCs, lymphocytes, etc. NK cells can not only directly recognize and kill target cells, but also secrete IFN-γ (52, 53). The killing activity of NK cells is not limited by the major histocompatibility complex, does not rely on antibodies, and can kill target cells without sensitization. They are activated by cytokines produced by DCs and macrophages, releasing IFN-γ, TNF-α, and perforin (49, 54). NK cells can also produce IL-10, which can inhibit the secretion of IL-12 by DCs and prevent infinite amplification of immune effects (55). Oral pathogenic bacteria were inoculated to induce experimental periodontitis in mice deficient for NK killer receptor NKp46 expression, indicating that NK cells may play an essential role in the pathogenesis of periodontitis (56, 57). The recognition of *T. gondii* profilin protein by TLR11 on the surface of DCs activates CCL2^+^, a monocyte chemotactic protein that binds to CCR2^+^ to activate monocytes (49). One vital role of monocytes is to reduce the number of *T. gondii* in the brain tissue. Experiments have found that a large number of CCR2^+^ monocytes infiltrate the brain tissue of *T. gondii*-infected mice, indicating that monocytes can migrate to the brain tissue after *T. gondii* infection to exert anti-infective effects (11). Macrophages are the main cell type invaded by *T. gondii* and they kill the parasite and process and present antigens after they are activated by IFN-γ (58). Macrophages in mice release reactive oxygen species or intermediates to kill parasites (59). There is experimental evidence that the main infiltrating cells in the affected gingival tissue of periodontitis patients are M1 macrophages and a small amount of M2 macrophages have been found to release the anti-inflammatory factor IL-10. Still, their levels are significantly lower than those of pro-inflammatory factors (60). When *T. gondii* invades the host, DCs play an essential role in coordinating the host’s innate immune response (61). Meanwhile, DCs are the main source of IL-12 production and can prevent *T. gondii* infection effectively (61). After exposure to microorganisms, gingival tissue cells secrete pro-inflammatory cytokines, and DCs are stimulated by these cytokines to induce the proliferation of Th2 and regulatory T cells. Th2 cells can promote the differentiation of M2 macrophages and secrete anti-inflammatory cytokines. Regulatory T cells can also secrete anti-inflammatory cytokines to alleviate inflammatory reactions. When DCs interact directly with microorganisms, they induce Th1 cell expansion, which promotes M1-type macrophage differentiation, secretes pro-inflammatory cytokines, and exacerbates the inflammatory response in periodontal tissue (62, 63). B cells also play an essential role in the immune protection induced by *T. gondii* vaccine. After immunizing B cell-deficient mice with attenuated *T. gondii* tachyzoites and then attacking them with a strong strain of *T. gondii*, the susceptibility of B cell-deficient mice is significantly higher than that of healthy mice (64). Studies have found that *in vivo* injection of B cell activating factor can alleviate the symptoms of periodontitis in mice (65).

**Figure 6:**
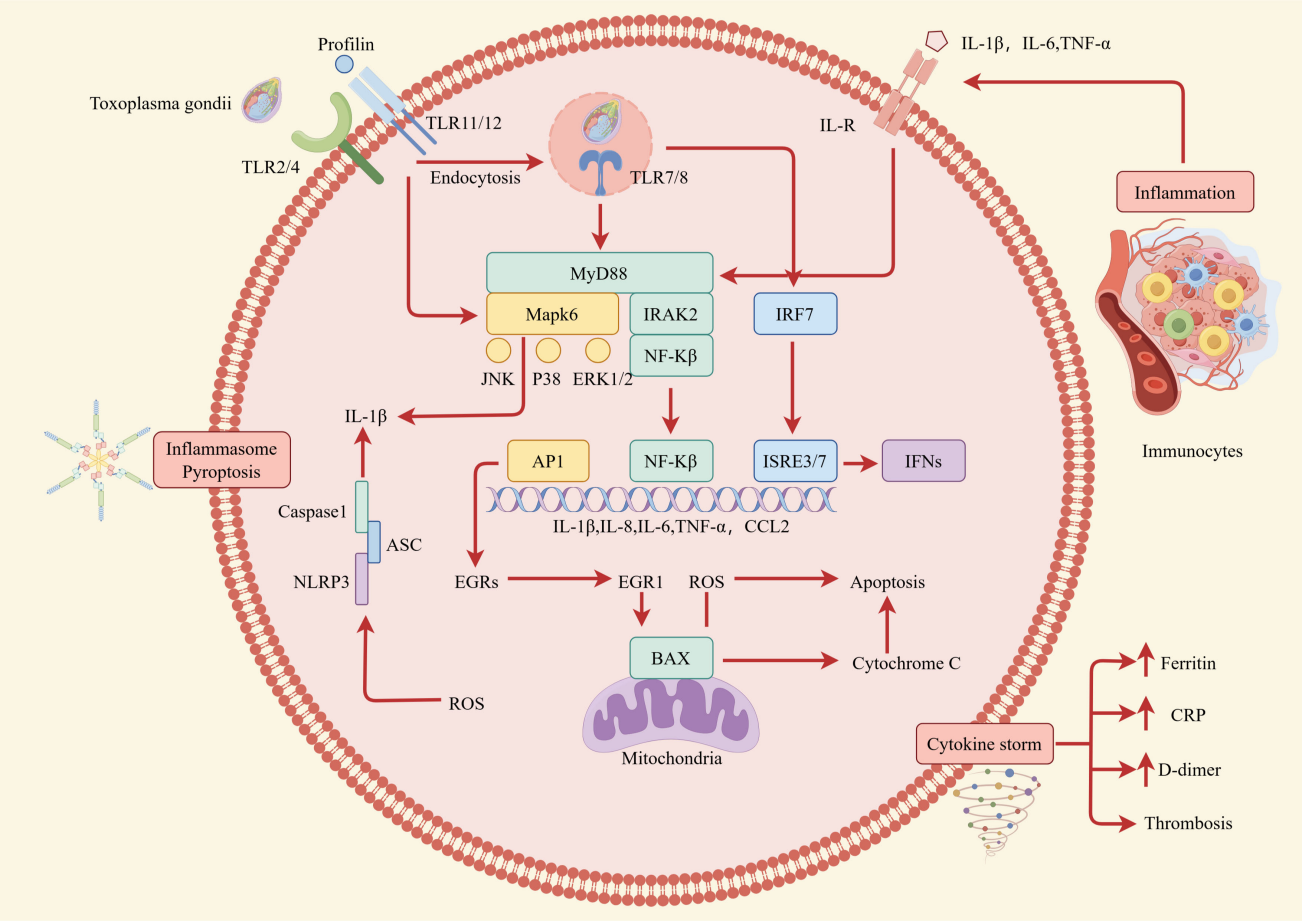
Intracellular immune response during *T. gondii* infection. The impact of cytokines on the host immune response is depicted at the level of intracellular interactions. Well-established pro-inflammatory cytokines such as IL-1, IL-6, and TNF-α are secreted by host cells and immunocytes after being stimulated by *T. gondii*. Then, by binding to their corresponding receptors, members of the IL-1, IL-6, and TNF-α families mainly activate transcription factors related to the secretion of pro-inflammatory cytokines and mediate the activation of immunocytes. This can lead to various cell fates, including pyroptosis and cytokine storm, both of which destroy periodontal tissue.

As shown in **Figure 6**, cytokines and their inducible products can not only control *T. gondii* infection but also participate in pathological damage. By binding to receptors, they degrade *T. gondii* antigens, regulate the differentiation of immune cells, and have an important impact on the entire process of the host’s immune system, leading to pathological damage of periodontal tissue. At present, research on cell signaling pathways and related molecules during the infection process of *T. gondii* is relatively limited, but an in-depth understanding of this aspect will have long-term significance in elucidating the invasion, parasitism, and interaction with the host of *T. gondii*. IL-12 is an important cytokine that activates the host’s immune response to *T. gondii*. It is mainly produced by DCs, monocytes, and neutrophils. In mice, CD8^+^ DCs are the main cell that produces IL-12 and TLR11, and TLR12 of DCs recognize and bind to the profilin protein of *T. gondii*, thus activating MyD88 in cells, initiating downstream signaling to produce IL-12 and regulate different types of cell activation during acute infection (51). In human, IL-12 is mainly produced by CD16^+^monocytes and CD1^+^DCs, but neither *T. gondii* profilin protein nor tachyzoite antigen can induce monocyte activation. The production of IL-12 depends on the endocytosis of monocytes (66). Due to the lack of TLR11 and TLR12 in human cells that recognize profilin proteins, the recognition of *T. gondii* nucleic acid is particularly important. Monocytes release *T. gondii* nucleic acids into the cytoplasm after endocytosis and thereby produce pro-inflammatory cytokines (67). In addition, IL-12 can also directly act on NK cells, significantly enhancing their killing ability and playing an essential role in the activation of NK cells during secondary infections (68, 69). Some clinical studies have shown a positive correlation between IL-12 levels and the severity of periodontal damage (70). Studies have shown that compared with the healthy control group, patients with severe periodontitis have higher levels of IL-12 in the gingival crevicular fluid and serum (71). Furthermore, IFN-γ is a common pro-inflammatory factor in periodontitis, which has been proven to be closely related to bone metabolism (72), and it is also an important cytokine to resist *T. gondii* infection (73). In human cells, the IFN-γ inducible indoleamine 2,3-dioxygenase 1 is an antimicrobial effector mechanism that limits pathogen proliferation *in vitro* (74). Studies show that IFN-γ is involved in alveolar bone loss in the ligature-induced periodontitis model, although it may inhibit osteoclast differentiation (75). In conclusion, the interplay between immune cells and cytokines in periodontitis contributes to the host’s defense against *T. gondii* while also potentially causing periodontal tissue damage.

## 8 Conclusions

The invasion of *T. gondii* into host cells is an active process, including adhesion, invasion of host cells, and the formation of a parasitophorous vacuole within host cells. This process is mainly related to proteins secreted by microtubules, rod-shaped bodies, and dense granules. However, the exact functions and mechanisms of certain functional molecules are not yet clear (40). Then, *T. gondii* disrupts the signaling cascade of host cells and spreads, especially within the host body. To survive in the host for a long time, *T. gondii* adjusts the host cell apoptosis program and cleverly responds to the host’s immune response. The host immune response is not entirely blocked, but only partially weakened. Multiple immune cells, such as neutrophils, DCs, monocytes, macrophages, NK cells, and lymphocytes, exert immune effects. They cooperate with each other and secrete various cytokines to resist *T. gondii* infection. Finally, the host immune response maintains a subtle balance between being induced and suppressed, enabling both host and parasite survival (51, 61). After the spread of *T. gondii* in the host body, it reaches different organs, causing eye toxoplasmosis, congenital Toxoplasmosis, encephalitis, and enteritis (59). Moreover, the inflammasome activation and neuronal injury caused by *T. gondii* infection can indirectly contribute to the pathogenesis of periodontal diseases by exacerbating chronic inflammation and dysregulated immune responses. These findings suggest that targeting the inflammasome pathway and addressing neuronal damage may offer new therapeutic strategies for managing periodontal diseases. The pathogenesis of these diseases needs further research. Although these organs have protective barriers, the host immune cells can reach these particular areas, and the mechanism needs further exploration. At present, research on the invasion of host periodontal tissue by *T. gondii*, as well as the pathogenic mechanism of *T. gondii* promoting the formation of periodontitis, is not yet clear. These studies have been conducted in animals and *in vitro* cells (10, 76), and whether there is a similar role in the pathogenic mechanism of *T. gondii* in humans remains to be confirmed. Although various immune cells and their secreted cytokines are able to exhibit immune responses against *T. gondii*, there are still many mechanisms between host immune response and parasitic infection that need further research. Our subsequent study will explore the correlation between parasites and periodontal diseases, providing a new perspective for the etiology of periodontal diseases research, and providing a new theoretical basis for the prevention, diagnosis, and treatment strategies of periodontal diseases.

**Figure 7:**
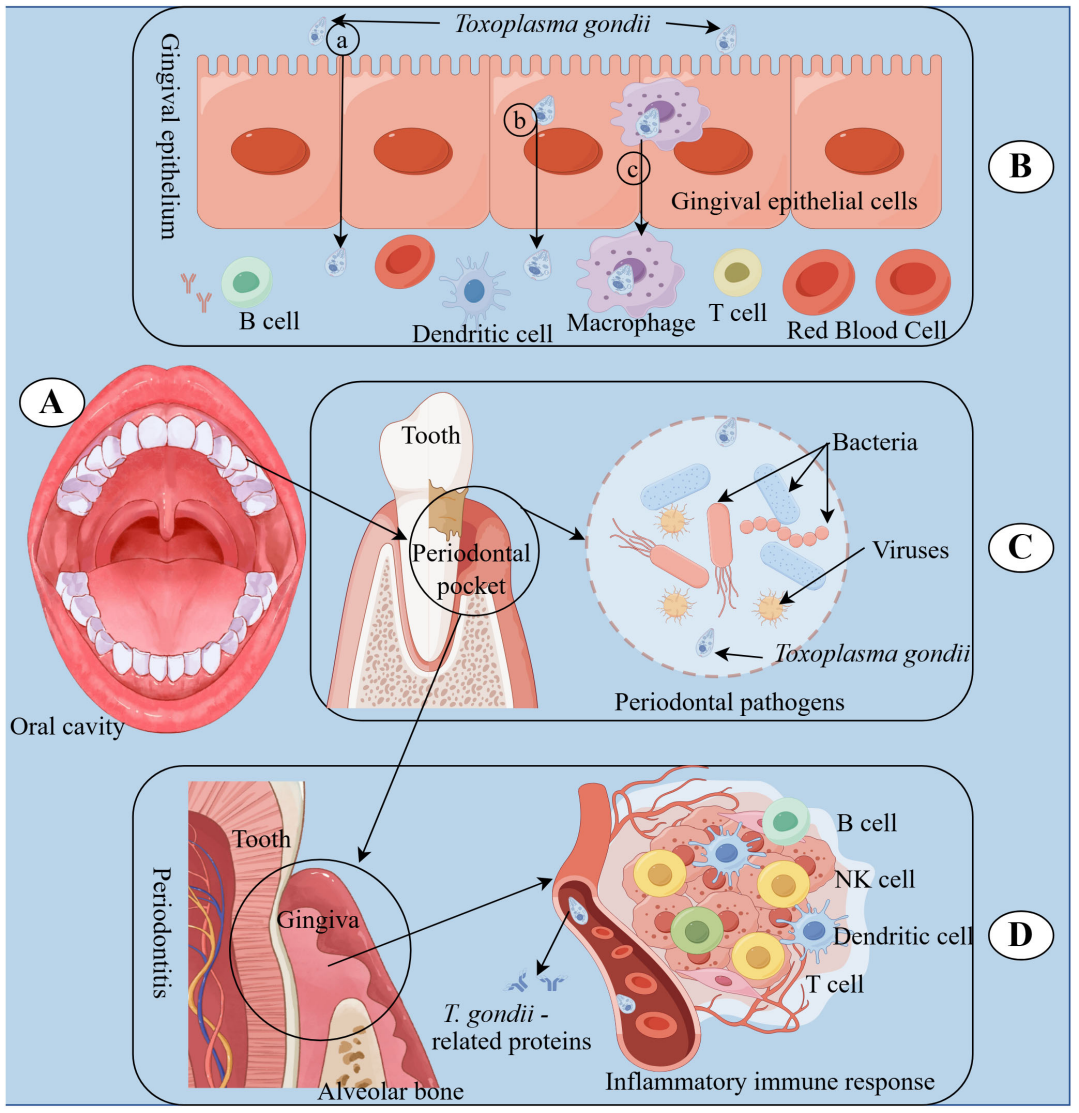
Underlying immune responses of *T. gondii* involvement in periodontal diseases. **(A)** The oral cavity is the starting place where *T. gondii* infection spreads. **(B)** *T. gondii* can enter the bloodstream through gingival epithelium on the inner wall of periodontal pockets using different pathways, including paracellular (a), transcellular (b), and “Trojan horse” (c) pathways, leading to dissemination and persistent infection within the host. **(C)** *T. gondii* can collaborate with periodontal pathogens in dental plaque, including bacteria and viruses, to cause periodontitis. **(D)** *T. gondii* can cause periodontitis by releasing *T. gondii*-related proteins and regulating host immune reactions.

In summary, our findings highlight the multifaceted role of *T. gondii* in the pathogenesis of periodontal diseases. As illustrated in **Figure 7**, *T. gondii* can initiate and exacerbate periodontal inflammation through various mechanisms. The oral cavity serves as the potential site for *T. gondii* infection, which can then spread throughout the body. *T. gondii* invades through ulcerated gingival surfaces using different pathways, including paracellular, transcellular, and “Trojan horse” pathways, to disseminate systemically and establish persistent infection (21, 77). The paracellular pathway involves *T. gondii* traversing tight junctions between adjacent cells. The transcellular pathway includes *T. gondii* entering and exiting from epithelial cells, effectively crossing the epithelial barrier. The “Trojan horse” pathway refers to *T. gondii* infecting immune cells, inhibiting the expression of anti-parasitic genes, and evading host immunity, thereby crossing the epithelial barrier. Besides, *T. gondii* gains access to the bloodstream through the periodontal pocket, contributing to local tissue damage in collaboration with dental plaque. Finally, *T. gondii* causes periodontitis by releasing *T. gondii*-related proteins and modulating host immune responses. These insights provide a comprehensive understanding of the complex interplay between *T. gondii* and the host’s immune system, paving the way for potential therapeutic interventions in periodontal diseases.
